# HAND2-AS1 Works as a ceRNA of miR-3118 to Suppress Proliferation and Migration in Breast Cancer by Upregulating PHLPP2

**DOI:** 10.1155/2020/8124570

**Published:** 2020-09-14

**Authors:** Guolei Dong, Xiaorui Wang, Yan Jia, Yongsheng Jia, Weipeng Zhao, Jin Zhang, Zhongsheng Tong

**Affiliations:** ^1^Department of Breast Oncology, Tianjin Medical University Cancer Institute and Hospital, National Clinical Research Center of Cancer, Key Laboratory of Breast Cancer Prevention and Therapy, Tianjin Medical University, Ministry of Education, Key Laboratory of Cancer Prevention and Therapy, Tianjin, Tianjin's Clinical Research Center for Cancer, Tianjin 300060, China; ^2^The 3rd Department of Breast Cancer, Tianjin Medical University Cancer Institute and Hospital, National Clinical Research Center of Cancer, Key Laboratory of Breast Cancer Prevention and Therapy, Tianjin Medical University, Ministry of Education, Key Laboratory of Cancer Prevention and Therapy, Tianjin, Tianjin's Clinical Research Center for Cancer, Tianjin 300060, China

## Abstract

Large quantities of long noncoding RNAs (lncRNAs) have been verified to exert vital functions in the process of breast cancer (BC). lncRNA heart and neural crest derivatives expressed 2-antisense RNA 1 (HAND2-AS1) was reported to suppress the development of several cancers. However, its detailed function in BC remained unclear. In the current study, HAND2-AS1 was discovered to be low expressed in BC cell lines, and overexpression of HAND2-AS1 could repress proliferation, migration, and invasion but facilitate apoptosis in BC cells. Moreover, HAND2-AS1 was found to act as a sponge of miR-3118 which was detected to be upregulated in BC cell lines. miR-3118 depletion could constrict the progression of BC. HAND-AS1 hindered the course of BC by reducing the expression of miR-3118. Besides, PHLPP2 was treated as a downstream target of miR-3118 under the selection of RNA pull-down assays. HAND2-AS1 inhibited the process of BC by enhancing expression of PHLPP2. In summary, our study testified that HAND2-AS1 suppressed BC growth by targeting the miR-3118/PHLPP2 axis, indicating that HAND2-AS1 could be regarded as a potential target for BC treatment.

## 1. Introduction

Breast cancer (BC) is a common cancer with high morbidity among females globally [1]. It was a great threat to female health and brought much trouble and disturbance to work and life. Great efforts were spared, and progress was achieved in treatment of BC [2]. It is extremely crucial to make an early prognosis for BC. Thus, it is necessary to have a thorough understanding of the molecular mechanism of BC.

Substantial long noncoding RNAs (lncRNAs) were reported to bring about vital effects on the progression of cancers [3, 4]. UCA1 strengthened resistance to tamoxifen in BC via activating the Pl3K/AKT signaling pathway [5]. FAGF13-AS1 was discovered to exert inhibitory effect on glycolysis and stemness in BC [6]. lncRNA heart and neural crest derivatives expressed 2-antisense RNA 1 (HAND2-AS1) was introduced to be downregulated in colorectal cancer [7], chronic myeloid leukemia [8], and non-small-cell lung cancer [9]. Nevertheless, its role has not been analyzed in BC.

Numerous essays acclaimed that the competing endogenous RNA (ceRNA) regulatory system played huge roles in regulating the mechanism of cancers [10, 11]. In this system, lncRNAs functioned as sponges of microRNAs (miRNAs), and their downstream target mRNAs were released to code into proteins [12, 13]. NEAT1 was identified as an oncogene in BC by targeting miR-448/ZEB1 [14]. LINC01116 is competitively bound to miR-145 to regulate the expression of ESR1 in BC [15]. In this study, we mainly studied the role of HAND2-AS1 in the ceRNA system.

miRNAs were a class of RNAs which were around 18-22 nucleotides in length without the capacity of coding proteins [16]. Emerging studies indicated that aberrant expression of miRNAs acted as oncogene or tumor inhibitors in cancers. miR-590-5p was reported as a tumor inhibitor in BC by interacting with SOX2 [17]. miR-198 exerted inhibitory effect on BC progression via targeting CUB [18]. Nonetheless, how miR-3188 took part in the BC progression remained unclear.

The main purpose of our current study was to explore the role of HAND2-AS1 in BC cells.

## 2. Results

### 2.1. Overexpression of HAND2-AS1 Repressed Proliferation, Migration, and Invasion in BC Cell

**Figure 1:**
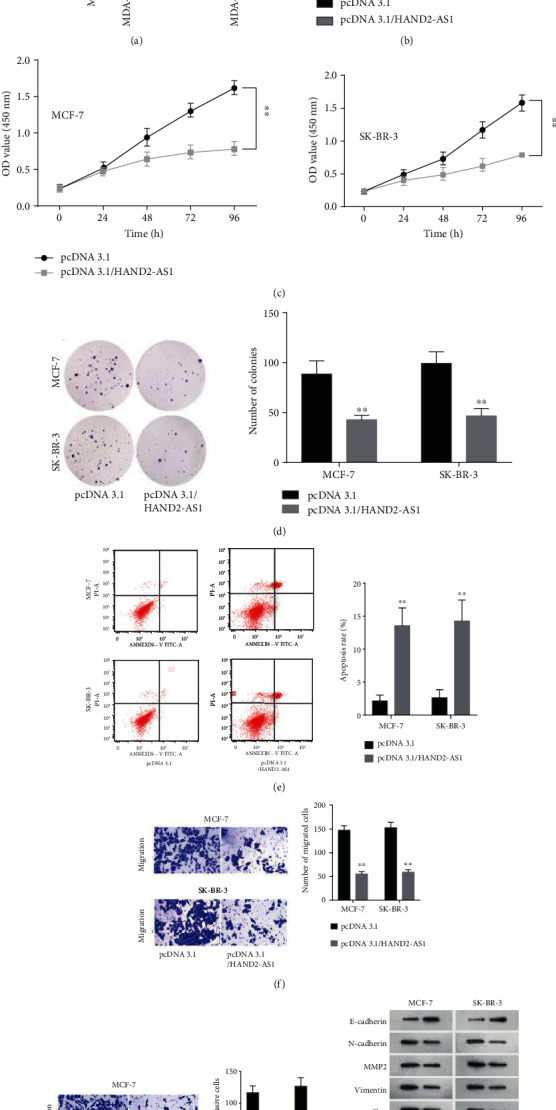
Overexpression of HAND2-AS1 repressed proliferation, migration, and invasion in BC cell. (a) RT-qPCR assays were constructed to evaluate expression of HAND2-AS1 in BC cell lines (MCF-7, MDA-MB-231, SK-BR-3, and MDA-MB-453) and normal mammary epithelial cell (MCF-10A). (b) RT-qPCR assays were performed to appraise the expression of HAND2-AS1 in cells transfected with pcDNA3.1/HAND2-AS1. (c, d) CCK8 and colony formation assays were conducted to appraise cell proliferation in cells transfected with pcDNA3.1/HAND2-AS1. (e) Flow cytometry analysis was performed to assess the apoptosis rate. (f, g) Transwell assays were carried out to assess the migration and invasion capacity. (h) Western blot was used to measure E-cadherin, N-cadherin, MMP2, Vimentin, and slug proteins.

To investigate the role of HAND2-AS1 in BC, we used RT-qPCR assays to determine the expression of HAND2-AS1 in BC cell lines (MCF-7, MDA-MB-231, SK-BR-3, and MDA-MB-453) and normal mammary epithelial cell (MCF-10A). The results showcased that HAND2-AS1 was at a low level of expression in BC cell lines in comparison with MCF-10A (Figure 1(a)). Then, pcDNA3.1/HAND2-AS1 was transfected into MCF-7 and SK-BR-3 cells. The outcomes disclosed that HAND2-AS1 expression was rose dramatically in comparison with the normal control (Figure 1(b)). To explore the influence of HAND2-AS1 on the development of BC, we conducted gain-of-function assays. The ability of proliferation assessed by CCK8 and colony formation assays was attenuated via overexpression of HAND2-AS1 (Figures 1(c) and 1(d)). The apoptosis rate was ascended by overexpression of HAND2-AS1 (Figure 1(e)). The capacities of migration and invasion were lessened by pcDNA3.1/HAND2-AS1 in transwell assays (Figures 1(f) and 1(g)). The proteins of the epithelial-mesenchymal transition (EMT) process were examined by western blot. The consequences depicted that E-cadherin protein was enhanced but N-cadherin, MMP2, Vimentin, and slug proteins were declined by overexpression of HAND2-AS1 (Figure 1(h)). In brief, HAND2-AS1 was low expressed in BC cell lines and overexpression of HAND2-AS1 inhibited the process of BC.

### 2.2. miR-3118 Depletion Inhibited BC Proliferation While Facilitating Apoptosis in BC

**Figure 2:**
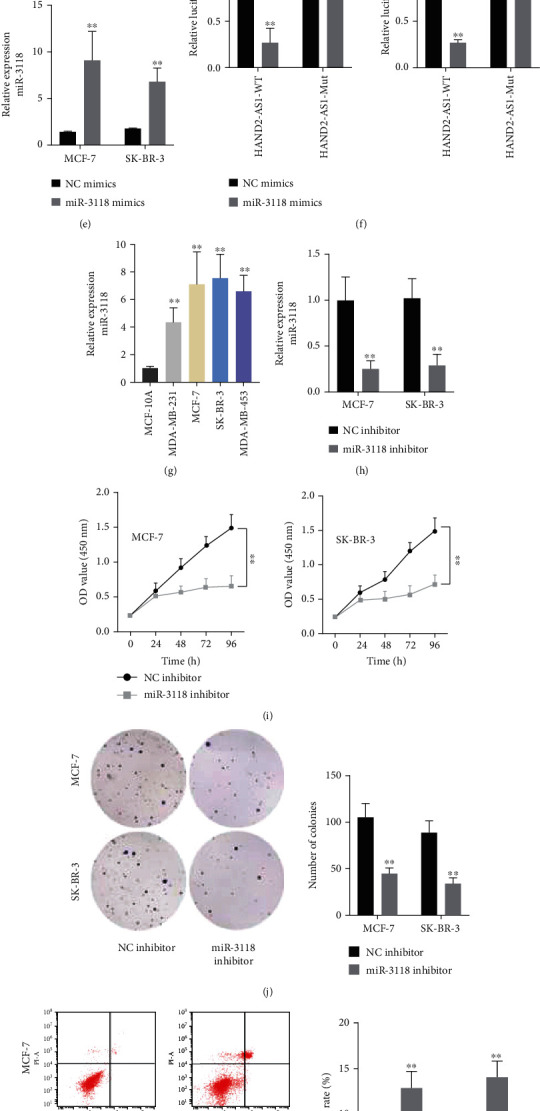
miR-3118 depletion inhibited BC proliferation while facilitating apoptosis in BC. (a) FISH assays were constructed to judge the subcellular localization of HAND2-AS1. (b) pcDNA3.1/HAND2-AS1 were transfected into cells, and the expression of miRNAs was detected in MCF-7 and SK-BR-3 cells. (c) The binding sequences between HAND2-AS1 and miR-3118 were projected by bioinformatics. (d) RNA pull-down assays were carried out to illustrate the combination between miR-3118 and HAND2-AS1. (e) miR-3118 mimics were transfected into cells, and the expression of miR-3118 was tested by RT-qPCR assays. (f) Luciferase reporter assays were conducted to demonstrate the combination between HAND2-AS1 and miR-3118. (g) The expression of miR-3118 was examined in BC cell lines by RT-qPCR assays. (h) miR-3118 expression was tested in cells transfected with miR-3118 inhibitor. (i, j) CCK8 and colony formation assays were applied to detect cell proliferation ability. (k) Flow cytometry analysis was conducted to probe the apoptosis rate. (l, m) Transwell assays were performed to estimate capacities of migration and invasion. (n) Western blot assays were conducted to measure associated proteins of the EMT process.

To analyze the role of HAND2-AS1 in the ceRNA regulatory system, we performed FISH assays, and the outcomes displayed that HAND2-AS1 accumulated in cytoplasm (Figure 2(a)). Subsequently, with the help of starBase, we discovered several miRNAs with binding sites for HAND2-AS1. These miRNA expressions were evaluated in cells transfected with pcDNA3.1/HAND2-AS1. The results disclosed that only miR-3118 expression was decreased distinctly in contrast with the control group (Figure 2(b)). The binding sequences were exhibited by bioinformatics (Figure 2(c)). RNA pull-down assays were carried out, and the outcomes delineated that miR-3118-WT with biotinylation could make HAND2-AS1 abundant but miR-3118-Mut with biotinylation could not (Figure 2(d)). We tested the expression of miR-3118 increased prominently by miR-3118 mimics in comparison with the control group (Figure 2(e)). The activity of plasmid built with HAND2-AS1-WT was declined remarkably by miR-3118 mimics, but there were no evident changes in that set with HAND2-AS1-Mut (Figure 2(f)). miR-3118 expression was appraised in BC cell lines, and it was showed that miR-3118 expression was extremely high in BC cell lines (Figure 2(g)). The expression of miR-3118 was found to be diminished in cells transfected with miR-3118 inhibitor (Figure 2(h)). Afterwards, we explored the effect of miR-3118 on the BC progression. miR-3118 inhibitor was transfected into cells. The results showcased that miR-3118 depletion could inhibit proliferation (Figures 2(i) and 2(j)) but foster the apoptosis rate (Figure 2(k)). And the capacity of migration and invasion was weakened by miR-3118 inhibitor and so did the EMT process (Figures 2(l)–2(n)). In short, miR-3118 was a target of HAND2-AS1, and depletion of miR-3118 could inhibit the process of BC.

### 2.3. HAND2-AS1 Restricted the Process of BC by Inhibiting miR-3118

**Figure 3:**
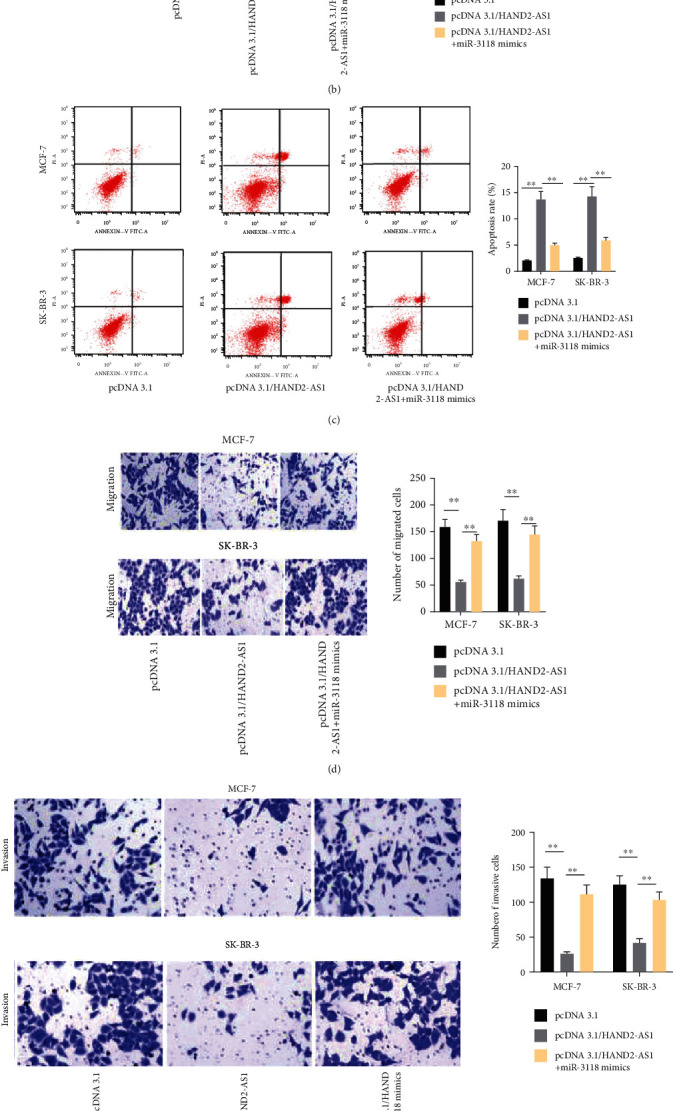
HAND2-AS1 restricted the process of BC by inhibiting miR-3118. (a, b) CCK8 and colony formation assays were performed to measure cell proliferation ability in cells transfected with pcDNA3.1/HAND2-AS1 and miR-3118 mimics. (c) The rate of flow cytometry analysis was set up to estimate the apoptosis rate. (d, e) Transwell assays were conducted to determine abilities of cell migration and invasion. (f) Western blot was carried out to evaluate the relevant proteins of the EMT process.

To find out how HAND2-AS1 regulated miR-3118 in BC, we conducted rescue assays. MCF-7 and SK-BR-3 cells were transfected with pcDNA3.1/HAND2-AS1 and miR-3188 mimics. The cell proliferation assessed by CCK8 and colony formation assays was declined by pcDNA3.1/HAND2-AS1, but miR-3118 mimics could rescue the results induced by HAND2-AS1 overexpression (Figures 3(a) and 3(b)). The increased rate of apoptosis induced by HAND2-AS1 overexpression could be rescued by miR-3118 mimics (Figure 3(c)). The lessened migration and invasion abilities assessed by transwell assays were counteracted by miR-3118 mimics (Figures 3(d) and 3(e)). Overexpression of HAND2-AS1 lifted the proteins of E-cadherin but decreased proteins of N-cadherin, MMP2, Vimentin, and slug, but miR-3118 mimics reversed the results made by HAND2-AS1 (Figure 3(f)). In conclusion, HAND2-AS1 inhibited the process of BC by downregulating miR-3118.

### 2.4. PHLPP2 Was a Downstream Target of miR-3118

**Figure 4:**
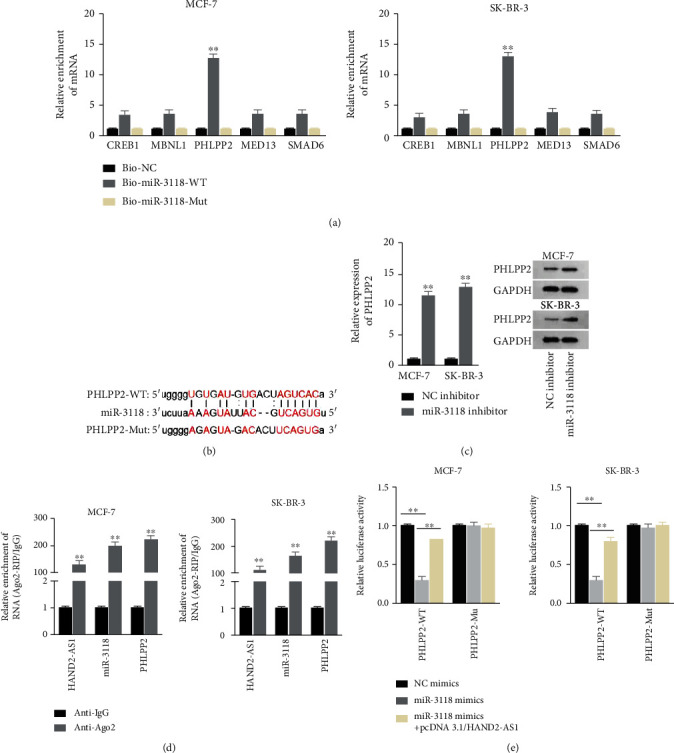
PHLPP2 was a downstream target of miR-3118. (a) RNA pull-down assays were carried out to verify which mRNA could bind to miR-3118. (b) The binding sites between miR-3118 and PHLPP2 were predicted by bioinformatics. (c) PHLPP2 expression and protein were tested in cells transfected with miR-3118 inhibitor by RT-qPCR and western blot. (d) RIP assays were performed to certify that HAND2-AS1, miR-3118, and PHLPP2 coexisted in RNA-induced silencing complexes (RISCs). (e) Luciferase reporter assays were conducted to testify the competing relationship between HAND2-AS1 and PHLPP2.

Then, we searched the starBase and found that several mRNAs had possibilities to bind to miR-3118. RNA pull-down assays were applied, and the results manifested that biotinylated miR-3118-WT only could make PHLPP2 abundant while no changes could be seen in other mRNAs (Figure 4(a)). The binding sites between miR-3118 and PHLPP2 were predicted by bioinformatics (Figure 4(b)). miR-3118 inhibitor was transfected into cells, and the expression of PHLPP2 and proteins were measured by RT-qPCR and western blot. The results depicted that both PHLPP2 expression and proteins were increased by miR-3118 inhibitor (Figure 4(c)). RIP assays were applied, and the results revealed that HAND2-AS1, PHLPP2, and miR-3118 were enriched in Ago2 antibody not in IgG antibody (Figure 4(d)). Luciferase reporter assays were conducted, and the results showed that miR-3118 mimics diminished the activity of plasmid constructed with PHLPP2-WT but not that set with PHLPP2-Mut. After transfecting pcDNA3.1/HAND2-AS1 into cells, the activity of plasmid built with PHLPP2-WT was recovered partially (Figure 4(e)). In a word, PHLPP2 was a downstream target of miR-3118.

### 2.5. HAND2-AS1 Hindered the Course of BC through Elevating PHLPP2 Expression

**Figure 5:**
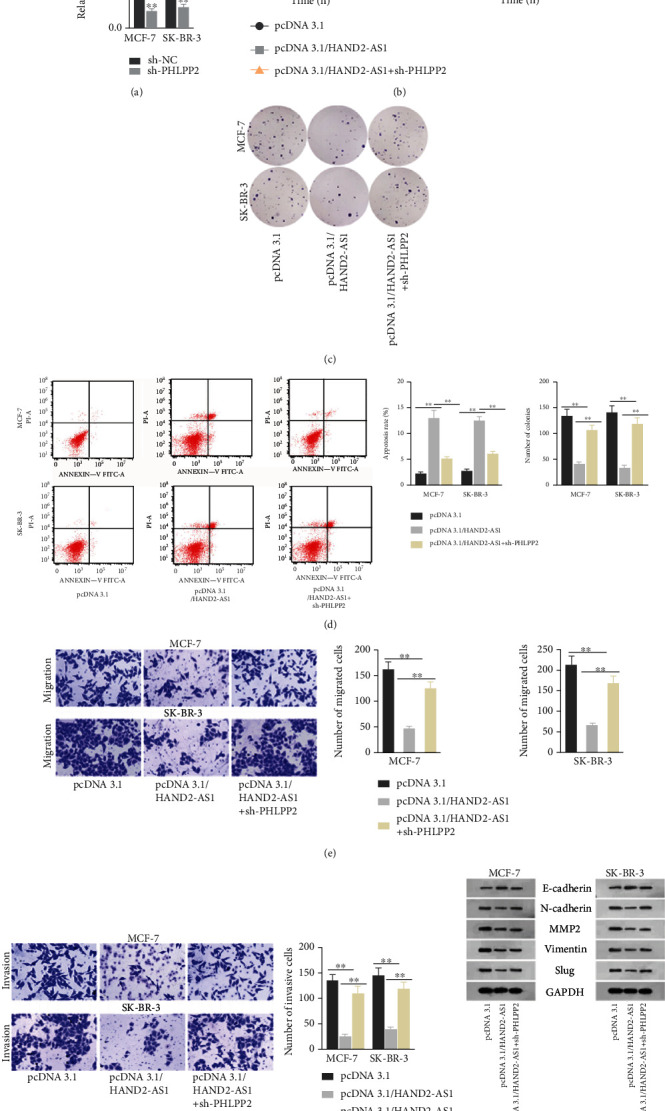
HAND2-AS1 hindered the course of BC through elevating PHLPP2 expression. (a) PHLPP2 expression was evaluated in cells transfected with pcDNA3.1/PHLPP2. (b, c) CCK8 and colony formation assays were carried out to estimate cell proliferation in cells transfected with pcDNA3.1/PHLPP2 and sh-PHLPP2. (d) The apoptosis rate was measured in flow cytometry analysis. (e, f) Transwell assays were conducted to evaluate capacity of migration and invasion. (g) Western blot was performed to measure relevant proteins of the EMT process.

To investigate how HAND2-AS1 mediated PHLPP2 in BC, we constructed rescue assays. Firstly, we examined the expression of HAND2-AS1 in cells transfected with sh-PHLPP2. The results revealed that PHLPP2 expression was cut down by sh-PHLPPP2 (Figure 5(a)). Then, pcDNA3.1/HAND2-AS1 and sh-PHLPP2 were transfected into cells. The results delineated that knockdown of PHLPP2 could rescue the effects of HAND2-AS1 overexpression on proliferation, apoptosis, migration, and invasion (Figures 5(b)–5(f)). The tendencies of relevant proteins induced by HAND2-AS1 overexpression in the EMT process were reversed by knockdown of PHLPP2 as well (Figure 5(g)). In summary, HAND2-AS1 could inhibit the progression of BC by stimulating the degradation of PHLPP2.

### 2.6. Overexpression of HAND2-AS1 Constricted the Growth of BC In Vivo

**Figure 6:**
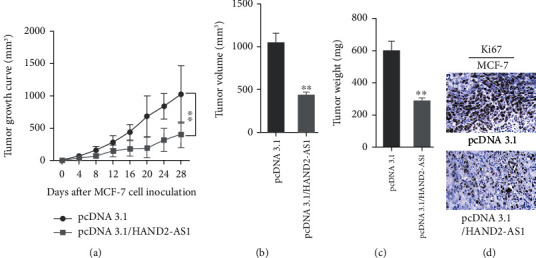
Overexpression of HAND2-AS1 constricted the growth of BC in vivo. (a–c) Tumor growth curve, volume, and weight were recorded and calculated in cells transfected with pcDNA3.1 and pcDNA3.1/HAND2-AS1. (d) Immunohistochemistry was conducted to examine the expression of Ki67 in cells transfected with pcDNA3.1 and pcDNA3.1/HAND2-AS1.

The effects of HAND2-AS1 on BC growth were verified in vivo. MCF-7 cells were transfected into pcDNA3.1/HAND2-AS1 and pcDNA3.1 vector. And the cells were injected into nude mice. After culture for 28 days, the process of tumor growth was recorded. The tumors transfected with pcDNA3.1/HAND2-AS1 grew slower than that with pcDNA3.1 vector (Figure 6(a)). The tumors were taken out, and both volume and weight of tumors with pcDNA3.1/HAND2-AS1 were smaller than that with pcDNA3.1 vector (Figures 6(b) and 6(c)). Immunohistochemistry was conducted to examine the expression of Ki67. The results revealed that Ki67 expression was declined conspicuously by pcDNA3.1/HAND2-AS1 compared with the normal control (Figure 6(d)). Collectively, overexpression of HAND2-AS1 could repress tumor growth in vivo.

## 3. Discussion

In this study, we mainly analyzed HAND2-AS1 and discovered that it is downregulated in BC cell lines, which was in accordance with previous reports [19, 20]. In addition, HAND2-AS1 could suppress proliferation, migration, and invasion but accelerated death in BC cells, which was consistent with the findings in the previous study about the HAND2-AS1 role in cancers [20, 21]. In addition, there has not been research involving the effect of HAND2-AS1 in a breast cancer animal model. Therefore, we conducted experiments in vivo to disclose the influence of HAND2-AS1 upregulation on the progression of BC malignancy. The tumor volume in the mouse group with pcDNA3.1/HAND2-AS1 was smaller than that in the normal group. So was the tumor weight, supporting that HAND2-AS1 works as an inhibitor in breast cancer.

Previous studies demonstrated that lncRNAs sponged miRNAs to regulate mRNA expression and thus the progression of cancers in the ceRNA regulatory system [22, 23]. Specifically, HAND2-AS1 was reported to sponge miR-340-5p to upregulate BCL2L11, inducing cell apoptosis in BC [24]. In this research, we discovered HAND2-AS1 targeted and inhibited miR-3118 in BC cell lines. Also, miR-3118 depletion repressed proliferative, invasive, and migratory capacities and promoted apoptosis of BC cells. The rescue assays demonstrated that HAND2-AS1 could hinder the process of BC by suppressing miR-3118 expression.

PHLPP2 was unveiled as a tumor suppressor in multiple cancers acting as an inhibitor of AKT and inducing apoptosis of cancer cells [25, 26]. On the other hand, microRNAs, lncRNAs, or other exogenous molecules interact with PHLPP2, thus modulating the cellular functions through the AKT pathway in tumors and diseases [27–30]. In our study, we selected out PHLPP2 by RNA pull-down assays from several mRNAs targeting miR-3118. Then, we knocked down the PHLPP2 in BC cells and discovered that inhibition of PHLPP2 greatly reversed the inhibitory impact on cell functions brought by HAND2-AS1. Taken together, this study showed that HAND2-AS1 sponges and inhibits miR-3118, curbing cell proliferation, migration, and invasion and inducing apoptosis of BC cells. Besides, miR-3118 suppressed PHLPP2 by targeting it while silencing PHLPP2 could alleviate the impact of HAND2-AS1 in BC. Therefore, we presented that HAND2-AS1 competed binding to miR-3188 with PHLPP2 and curbed the progression of breast cancer in vitro by elevating PHLPP2. In addition, in vivo assays also verified the suppressive effect of HAND2-AS1 in BC. However, there is one shortcoming in this present study. The subcutaneous tumor models are not sufficient for understanding of pathogenesis of breast cancer. Therefore, further tumor models are required to validate the role of HAND2-AS1 in the progression of breast cancer.

To sum up, our studies confirmed that HAND2-AS1 could inhibit BC proliferation, migration, and invasion and induce apoptosis as a ceRNA of miR-3188 to elevate PHLPP2 expression, which poses a potential that HAND2-AS1/miR-3188/PHLPP2 might be a therapeutic axis for breast cancer in the future.

## 4. Materials and Methods

### 4.1. Cell Lines and Transfection

BC cell lines (MDA-MB-231, MCF-7, SK-BR-3, and MDA-MB-453) and human mammary epithelial cell line (MCF10A) were both procured from the Cell Bank of the Chinese Academy of Sciences (Shanghai, China). DMEM, 10% fetal bovine serum (FBS), and 100 U/L penicillin/streptomycin were bought from Gibco (Grand Island, NY, USA) for cell culture in 5% CO_2_ at 37°C. MCF-7 and SK-BR-3 cells in 6-well plates (1 × 10^5^ cells/well) were prepared for 48 h of transfection utilizing Lipofectamine 2000 to differentiate the expression levels of HAND2-AS1, miR-3118, and PHLPP2 in cells (Invitrogen, Carlsbad, CA, USA). The overexpression plasmids pcDNA3.1/HAND2-AS1 and control (pcDNA3.1), miR-3118 mimics and NC mimics, as well as the silencing plasmids miR-3118 inhibitor and NC inhibitor, and sh-PHLPP2 and control (sh-NC) were all processed by RiboBio (Guangzhou, China).

### 4.2. Real-Time qPCR (RT-qPCR)

Total cellular RNAs were extracted from cell samples via the TRIzol reagent (Invitrogen) to synthesize cDNA with the PrimeScript RT reagent kit (Takara Biotechnology, Tokyo, Japan). qPCR was developed using SYBR Green PCR Master Mix (Invitrogen) on a Bio-Rad IQ5 thermocycler (Bio-Rad Laboratories, Inc., Hercules, CA, USA). RNA levels of HAND2-AS1, miR-3118, and PHLPP2 in all groups were normalized to U6 or GAPDH using the 2^-*ΔΔ*CT^ method.

### 4.3. Cell Counting Kit-8 (CCK8)

BC cells in 96-well plates (5 × 10^3^ cells/well) were incubated with CCK8 solution (Beyotime, Shanghai, China) in a 37°C, 5% CO_2_ incubator for 48 h. Cell viability in different groups was monitored by assessing the optical density by a microplate reader at absorbance of 450 nm.

### 4.4. Colony Formation Assay

Clonogenic cells of MCF-7 and SK-BR-3 were incubated in 6-well plates (1 × 10^3^ cells/well), following 14 days of incubation. Cells were stained with 0.5% crystal violet in 4% paraformaldehyde, and colonies were counted in all groups as a reference to the cell proliferation capability.

### 4.5. Cell Apoptosis Assay

BC cells were first washed in cold phosphate-buffered saline (PBS). Then, they were resuspended in 100 mL of binding buffer. 5 mL of Annexin V-FITC (Invitrogen) was added for 15 min in the dark at room temperature. Then, 5 mL of propidium iodide (PI; Invitrogen) was added for 15 min, followed by analysis of flow cytometry (BD Biosciences, San Jose, CA, USA). The flow cytometry assays analyzed changes in apoptosis rates in all groups.

### 4.6. Transwell Assay

Transfected BC cells were collected and incubated in serum-free medium. The 24-well transwell chamber (Costar, MA, USA) with matrigel coating was for invasion or migration assay. BC cells were put into the upper compartment, while the lower compartment was filled with complete culture medium. After 48 h of incubation, invasive and migratory cells were fixed and stained with 4% paraformaldehyde and 0.1% crystal violet for counting. The number of invaded or migrated cells was counted and served as a reference to cell migration or invasion ability in different groups.

### 4.7. Western Blotting

The western blotting method was used to observe the relative protein expression in cells of all groups. Protein samples from MCF-7 and SK-BR-3 cells were separated by electrophoresis on 10% SDS polyacrylamide gels and transferred onto PVDF membranes which were then blocked with 5% skimmed milk at room temperature for 2 h. The incubation with primary antibodies against E-cadherin, N-cadherin, MMP2, Vimentin, slug, PHLPP2, and GAPDH, along with the corresponding secondary antibodies (all from Abcam, Cambridge, MA, USA), was performed prior to analysis of the enhanced chemiluminescence reagent (Santa Cruz Biotechnology, Santa Cruz, CA, USA).

### 4.8. FISH Assay

To investigate the localization of HAND1-AS1 in cells, we resorted to the FISH method. For the FISH assay, the HAND2-AS1 RNA probe was produced by RiboBio. After treating MCF-7 and SK-BR-3 cells with Hoechst, stained cells were photographed by a laser scanning confocal microscope (ZEISS, Jena, Germany).

### 4.9. RNA Pull-Down Assay

To investigate the interactions between RNAs involved, we performed RNA pull-down assay. The miR-3118-WT, miR-3118-Mut, and NC were biotin-labeled to Bio-miR-3118-WT, Bio-miR-3118-Mut, and Bio-NC by RiboBio, then incubated with cell lysates and Dyna-beads (Invitrogen). After washing, the bound RNAs were assayed by RT-qPCR.

### 4.10. Bioinformatics Analysis and Dual-Luciferase Reporter Assays

StarBase (http://starbase.sysu.edu.cn/agoClipRNA.php?source=lncRNA) was used online to predict the binding sites between HAND2-AS1 and miR-3118 as well as miR-3118 and PHLPP2. To confirm the bindings between HAND2-AS1 and miR-3118, and miR-3118 and PHLPP2, we did the dual-luciferase reporter assays. The pmirGLO Vector (Promega, Madison, WI, USA) containing the wild-type (WT) and mutant (Mut) miR-3118 binding sites within HAND2-AS1 sequence or PHLPP2 3′-UTR were designed and termed as HAND2-AS1-WT/Mut and PHLPP2 WT/Mut. The indicated transfection plasmids were cotransfected into BC cells with HAND2-AS1-WT/Mut or PHLPP2 WT/Mut for 48 h, then examined by the Dual-Luciferase Assay System (Promega).

### 4.11. RNA Immunoprecipitation (RIP)

In light of the guidebook of Magna RIP™ RNA-Binding Protein Immunoprecipitation Kit (Millipore, Bedford, MA, USA), 1 × 10^7^ BC cells in RIP lysis buffer were subjected to immunoprecipitation with antibodies against Ago2 or control IgG (Millipore). After adding beads, the precipitates were assayed by RT-qPCR. The RIP method was used to assess the RNA enrichment in cells.

### 4.12. In Vivo Assay

6 weeks old of male nude mice were procured from the National Laboratory Animal Center (Beijing, China) and maintained in SPF animal laboratory, with the approval of the Animal Research Ethics Committee of Tianjin Medical University Cancer Institute and Hospital. MCF-7 cells transfected with pcDNA 3.1/HAND2-AS1 or pcDNA 3.1 were injected to mice subcutaneously for 28 days to form in vivo models of breast cancer, with tumor volume recorded every 4 days. The tumor samples from killed mice were weighed for further analysis.

### 4.13. Immunohistochemistry (IHC)

The tumor tissue samples from in vivo study were fixed by 4% paraformaldehyde and embedded in paraffin. After cutting, the sections of 4 *μ*m thick were subjected to IHC using anti-Ki67 (Santa Cruz Biotechnology), through which we evaluated the proliferation marker Ki67 in tumor tissues from the pcDNA 3.1/HAND2-AS1 group with the pcDNA 3.1 group as a control.

### 4.14. Statistical Analysis

All assays included three biological repeats. Data were processed by Student's test to compare between two groups, and ANOVA (one-way) was adopted when three or more groups were included in data in GraphPad PRISM 6 (GraphPad, San Diego, CA, USA), with *p* < 0.05 as the cutoff value.

## Data Availability

The data used to support the findings of this study are available from the corresponding authors upon request.
